# Neuromodulation Induced by Sitagliptin: A New Strategy for Treating Diabetic Retinopathy

**DOI:** 10.3390/biomedicines9121772

**Published:** 2021-11-26

**Authors:** Hugo Ramos, Patricia Bogdanov, David Sabater, Jordi Huerta, Marta Valeri, Cristina Hernández, Rafael Simó

**Affiliations:** 1 Diabetes and Metabolism Research Unit, Vall d’Hebron Research Institute, Universitat Autònoma de Barcelona, 08035 Barcelona, Spain; hugo.ramos@vhir.org (H.R.); patricia.bogdanov@vhir.org (P.B.); david.sabater@vhir.org (D.S.); jordi.huerta@vhir.org (J.H.); 2 Centro de Investigación Biomédica en Red de Diabetes y Enfermedades Metabólicas Asociadas (CIBERDEM), Instituto de Salud Carlos III (ICSIII), 28029 Madrid, Spain; 3 Unit of High Technology, Vall d’Hebron Research Institute, 08035 Barcelona, Spain; marta.valeri@vhir.org

**Keywords:** diabetic retinopathy, retinal neurodegeneration, dipeptidyl peptidase 4 inhibitors, sitagliptin, presynaptic proteins, db/db mouse model

## Abstract

Diabetic retinopathy (DR) involves progressive neurovascular degeneration of the retina. Reduction in synaptic protein expression has been observed in retinas from several diabetic animal models and human retinas. We previously reported that the topical administration (eye drops) of sitagliptin, a dipeptidyl peptidase-4 (DPP-4) inhibitor, prevented retinal neurodegeneration induced by diabetes in db/db mice. The aim of the present study is to examine whether the modulation of presynaptic proteins is a mechanism involved in the neuroprotective effect of sitagliptin. For this purpose, 12 db/db mice, aged 12 weeks, received a topical administration of sitagliptin (5 μL; concentration: 10 mg/mL) twice per day for 2 weeks, while other 12 db/db mice were treated with vehicle (5 μL). Twelve non-diabetic mice (db/+) were used as a control group. Protein levels were assessed by western blot and immunohistochemistry (IHC), and mRNA levels were evaluated by reverse transcription polymerase chain reaction (RT-PCR). Our results revealed a downregulation (protein and mRNA levels) of several presynaptic proteins such as synapsin I (*Syn1)*, synaptophysin (*Syp*), synaptotagmin (*Syt1)*, syntaxin 1A (*Stx1a)*, vesicle-associated membrane protein 2 (*Vamp2*), and synaptosomal-associated protein of 25 kDa (*Snap25*) in diabetic mice treated with vehicle in comparison with non-diabetic mice. These proteins are involved in vesicle biogenesis, mobilization and docking, membrane fusion and recycling, and synaptic neurotransmission. Sitagliptin was able to significantly prevent the downregulation of all these proteins. We conclude that sitagliptin exerts beneficial effects in the retinas of db/db mice by preventing the downregulation of crucial presynaptic proteins. These neuroprotective effects open a new avenue for treating DR as well other retinal diseases in which neurodegeneration/synaptic abnormalities play a relevant role.

## 1. Introduction

The concept of diabetic retina (DR) as a microvascular disease has evolved into that of a more complex diabetic complication in which neurodegeneration plays a significant role [1,2]. Thus, DR is now defined as a highly tissue-specific neurovascular complication [3]. Neurodegeneration and glial activation are primary events in the pathogenesis of DR and have been observed to occur before overt microangiopathy in experimental models of DR and in the retina of diabetic donors [4,5,6].

Neuronal damage associated with DR is not limited to cell apoptosis and glial activation but also includes other changes such as a reduction in synaptic protein expression [7,8]. In this regard, there is evidence that diabetes reduces the retinal content of several presynaptic proteins critical to the exocytosis of neurotransmitters and synaptic maintenance in murine models [8,9,10]. In postmortem human retinas, it has been reported that the protein content of presynaptic was depleted compared to non-diabetic donors [11]. Therefore, to prevent or ameliorate the downregulation of presynaptic proteins induced by diabetes would be a desirable effect of any neuroprotective drug.

We have previously demonstrated that topical administration of sitagliptin, a DPP-4 inhibitor, is effective in preventing retinal neurodegeneration (glial activation and neuronal apoptosis), as well as vascular leakage in db/db mouse model [12]. On this basis, the aim of the present study is to examine in an experimental model of diabetes (db/db mice) whether the modulation of presynaptic proteins is a mechanism involved in the neuroprotective effect of sitagliptin.

## 2. Materials and Methods

### 2.1. Animals

A total of 24 diabetic male db/db (BKS.Cg-Dock7m +/+ Leprdb/J) mice and 12 non-diabetic mice (db/+; (BKS.Cg-Dock7m + Leprdb/+) aged 7 weeks were obtained from Charles River Laboratories Inc (Calco Italy for the study). We have characterized the neurodegenerative process that occurs in the retinas of db/db mice, and we found that db/db mouse reproduces the features of the neurodegenerative process that occurs in the human diabetic retina [13]. Animals were bred and maintained at VHIR’s animal facility. Animals had free access to ad libitum food (ENVIGO Global Diet Complete Feed for Rodents, Mucedola, Milan, Italy) and filtered water. In order to minimize variability, animals were randomly housed (block randomization) in groups of 2 mice per cage, in Tecniplast GM-500 cages under standard laboratory conditions at 22 ± 2 °C, with 12 h light/dark cycle and relative humidity of 50–60%. Each cage held absorbent bedding and nesting material (BioFresh Performance Bedding 1/800 Pelleted Cellulose, Absorption Corp., Ferndale, WA, USA). Blood glucose levels were weekly measured through tail vein sampling (glucose assay kit; Abbott, Chicago, IL, USA).

All performed experiments with animals were conducted in compliance with European Community (86/609/CEE) and ARVO (Association for Research in Vision and Ophthalmology) tenets for the use of laboratory animals. This study was approved by the Animal Care and Use Committee of Vall d’Hebron Research Institute (VHIR) (Passeig de la Vall d’Hebron 119–129, Barcelona, Spain. Approval code 75/15, approval date 2 December 2015).

### 2.2. Topical Ocular Treatment

Sitagliptin [sitagliptin phosphate monohydrate (Y0001812, Merck KGaA, Darmstadt, Germany) eyedrops (10 mg/mL; 5 µL; n = 12) or vehicle [phosphate buffered saline (PBS)] eyedrops (5 µL; n = 12) were randomly administered directly onto the superior corneal surface of each eye using a micropipette in 10-week-old mice. The treatment (sitagliptin or vehicle) was administered twice daily for 15 days. Non-diabetic mice (n = 12) matched by age were used as control group. On day 15, the drop of sitagliptin or vehicle was administered animals 1h before euthanasia.

### 2.3. Electroretinogram (ERG)

Full field electroretinography (ERG) recordings were estimated using the Ganzfeld ERG platform (Phoenix Research Laboratories, Pleasanton, CA, USA) following ISCEV (International Society for Clinical Electrophysiology of Vision) recommendations [14]. Animals were dark adapted for at least 8 h prior to ERG recording and then anesthetized with isoflurane. Tropicamide (1%) was utilized to each eye prior to the test. A cutaneous ground electrode was placed near the base of the tail, a needle electrode was placed on the head between the two eyes, and a corneal electrode was placed near each eye. Carboxymethylcellulose (1%) drops were applied to the interior surface of the contact lens electrodes prior to their placement on the eyes. Three ERG components were assessed in terms of amplitude and timing.

### 2.4. Retinal Tissue Processing

On day 15, each animal was transcardially perfused with paraformaldehyde 4% (sc-281692, Santa Cruz Biotechnology, Dallas, TX, USA). Previously they received a 200 µL intraperitoneal injection of anesthesia, prepared with a mix containing 1 mL ketamine (GmbH, Hameln, Germany) and 0.3 mL xylazine (Laboratorios Calier S.A., Barcelona, Spain). Eyes were rapidly enucleated and for mRNA and protein evaluations the retinas were separated instantly after enucleation, frozen in liquid nitrogen, and stored at −80 °C. For RNA extraction, the retinas were introduced in individual tubes with 140 µL of TRIzol reagent (15596018, Invitrogen^TM^, Carlsbad, CA, USA). For Western Blot assays, retinas were directly dispensed in an empty tube until protein extraction. Finally, for immunohistochemistry assessment, eyes were not dissected after enucleation and were fixed in 4% paraformaldehyde during 5 h before paraffin embedding. Sections were mounted on slides and stored at 4 °C.

#### 2.4.1. RNA Extraction and Quantitative Reverse Transcription Polymerase Chain Reaction (RT-PCR) Assay

Neuroretinas (previously stored at −80 °C in TRIzol) were treated with DNase (18068015, ThermoFisher Scientific, Waltham, MA, USA) to eliminate genomic contamination and were purified on RNeasy MinElute column (74106, Qiagen, Hilden, Germany). After supernatant removal, RNA sediment was obtained and resuspended in 30 µL of RNAse free water (AM9937, ThermoFisher Scientific, Waltham, MA, USA). An Agilent 2100 Bioanalyzer and a Nanodrop spectrophotometer were used for samples integrity and quantity respectively. cDNA reverse transcription was performed in a T100 Thermal Cycler (Bio-rad, Hercules, CA, USA) using High-Capacity cDNA Reverse Transcription Kit (4368814, ThermoFisher Scientific, Waltham, MA, USA) and Oligo(dT)18 Primer (SO131, ThermoFisher Scientific, Waltham, MA, USA). RT-PCR was carried out using SYBR Green PCR Master Mix (4309155, Applied Biosystems, Warrington, UK) and a 7.900 HT Sequence Detection System in 384-well optical plates with specific primers (displayed in Table 1). Relative quantities were calculated using the ABI SDS 2.4 RQ software and presented as a ratio between them and the endogenous controls (*B2m* and *Actin B (Actb)*).

#### 2.4.2. Western Blotting

Protein extraction consisted in a sonication of neuroretinas for 10–15 s in 80 µL of lysis buffer [phenylmethanesulfonylfluoride (PMSF), 1mM; NaF, 100mM; Na3VO4 2mM; RIPA buffer (R0278, Sigma-Aldrich, St Louis, MO, USA); 1x protease inhibitor cocktail (P8340, Sigma-Aldrich, St. Louis, MO, USA)]. 25 µg of extracted protein were loaded in a 4–20% (vol./vol.) precast gels for SDS-PAGE (4561096, Bio-rad, Hercules, CA, USA), and electrophoresis was carried out at 100 V for 90 min. Proteins were then transferred to a polyvinylidene difluoride (PVDF) membrane (1620177, Bio-Rad Laboratories, Hercules, CA, USA) at 400 mA for 90 min and blocked in 5% powder milk (Central Lechera Asturiana, Spain) in 0.1% TBS-Tween. Primary antibodies (Table 2) were overnight incubated at 4 °C. On the next day, secondary antibodies (goat anti-rabbit and goat anti-mouse (Dako Agilent, Santa Clara, CA, USA)) were applied (1:10,000). Immunoreactive bands were detected using a WesternBright ECL HRP substrate kit (K-12045-D50, Advansta, CA, USA). Anti-vinculin (1:7.000, sc-73614; Santa Cruz, Dallas, TX, USA) was used as housekeeping to normalize protein levels. The densitometric analysis was assessed with Image J software (Version 1.8, National Institutes of Health, Bethesda, MD, USA).

#### 2.4.3. Immunofluorescence Analysis

Paraffin-embedded ocular globes were sectioned (3 µm) and mounted on 25.5 × 75.5 × 1.0 mm Poly-L-Lysine positive charged slides (S21.2113.A, Leica Biosystems, Wetzlar, Germany). Samples were deparaffinized in xylene, rehydrated in grade ethanol series, fixed in ice-cold acid methanol (−20 °C), and washed with PBS 0.01M at pH 7.4. Consecutively, eye sections were heated in a pressure cooker at 150 °C for 4 min in 250 mL of 1:10 diluted antigen retrieval with sodium citrate 10 mM, pH 6 (ab973, Abcam, Cambridge, UK). Then, sections were blocked with blocking solution (X0909, Dako Agilent, Santa Clara, CA, USA) for 1h at room temperature and then incubated overnight at 4 °C with specific primary antibodies (Table 3). The next day, after three washes in PBS, sections were incubated for 1 h in darkness with secondary antibodies (Alexa 488 and Alexa 594; 1/600, Molecular Probes). Samples were washed with PBS, counterstained with Hoechst 33342 (bisbenzimide) (14533, ThermoFisher Scientific, Waltham, MA, USA), and mounted with Prolong Mounting Medium Fluorescence (P36930, Prolong, Invitrogen^TM^, Thermo Fisher Scientific, Eugene, OR, USA) and a coverslip (15747592, ThermoFisher Scientific, Waltham, MA, USA). The images were obtained at a resolution of 1024 x 1024 pixels using laser confocal microscopy (Fluoview FV1000 Laser Scanning Confocal Microscope Olympus, Hamburg, Germany) and immunofluorescences were quantified with ImageJ software (U. S. National Institutes of Health, Bethesda, MD, USA).

#### 2.4.4. Statistical Analysis

Data are presented as mean ± SEM. Statistical comparisons were performed with Student unpaired test. When multiple comparisons were performed, one-way ANOVA followed by the Bonferroni test was used. Statistical significance was set at *p* < 0.05.

## 3. Results

**Figure 1:**
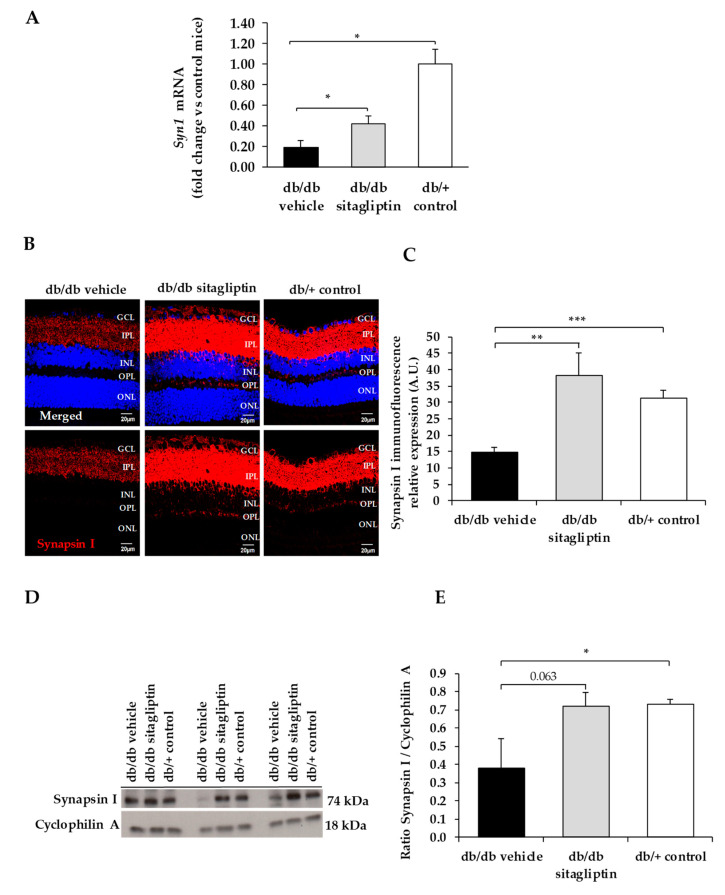
(**A**) Real-time quantitative RT-PCR analysis of synapsin I gene *(Syn1)* in db/db mice treated with vehicle (black bars), sitagliptin eye drops (grey bars), and in non-diabetic mice (white bars). (**B**) Immunofluorescence of synapsin I (red) among representative samples of diabetic retinas of experimental groups. Hoechst staining (blue) was used for nuclei labeling. ONL: outer nuclear layer; OPL: outer plexifom layer; INL: inner nuclear layer; IPL: inner plexiform layer; GCL: ganglion cell layer. Scale bars, 20 µm. (**C**) Quantification of synapsin I immunofluoresence in diabetic mice treated with vehicle eye drops (black bars), sitagliptin eye drops (grey bars), and in non-diabetic retinas (white bars). (**D**) Western blot bands of synapsin I and (**E**) Densitometric analysis in db/db mice treated with vehicle eye drops (black bars), sitagliptin eye drops (grey bars), and in non-diabetic mice retinas (white bars). Protein levels were normalized with cyclophilin A. * *p* < 0.05, ** *p* < 0.01, *** *p* < 0.001.

**Figure 2:**
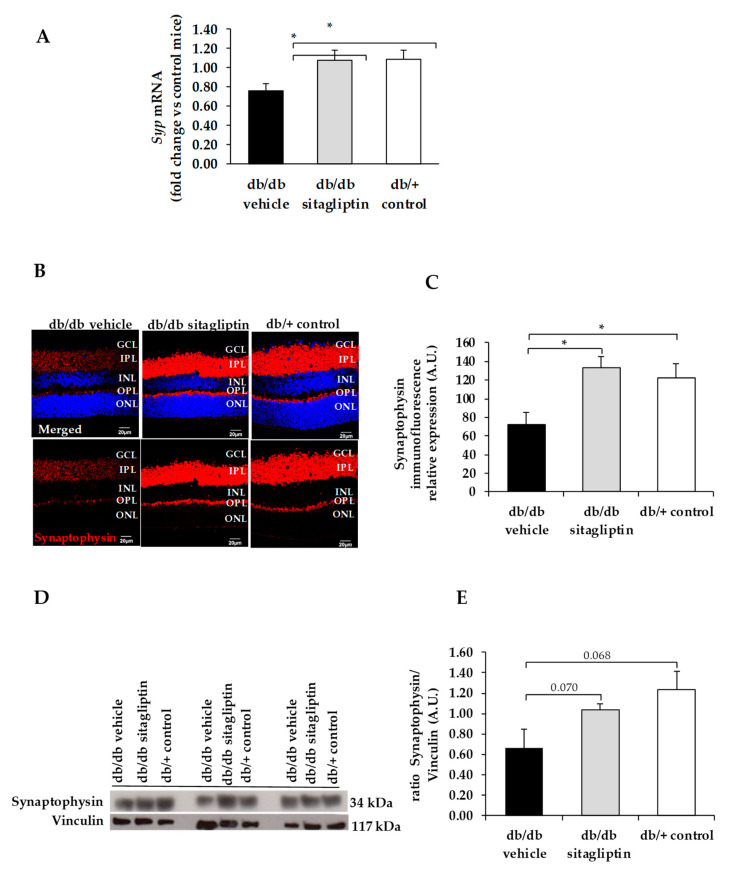
(**A**) Real-time quantitative RT-PCR analysis of synaptophysin gene *(Syp)* in db/db mice treated with vehicle (black bars), sitagliptin eye drops (grey bars), and in non-diabetic mice (white bars). (**B**) Immunofluorescence of synaptophysin (red) among representative samples of diabetic retinas of experimental groups. Hoechst staining (blue) was used for nuclei labeling. ONL: outer nuclear layer; OPL: outer plexifom layer; INL: inner nuclear layer; IPL: inner plexiform layer; GCL: ganglion cell layer. Scale bars, 20 µm. (**C**) Quantification of synaptophysin immunofluoresence in diabetic mice treated with vehicle eye drops (black bars), sitagliptin eye drops (grey bars), and in non-diabetic retinas (white bars). (**D**) Western blot bands of synaptophysin and (**E**) Densitometric analysis in db/db mice treated with vehicle eye drops (black bars), sitagliptin eye drops (grey bars), and in non-diabetic mice retinas (white bars). Protein levels were normalized with vinculin. * *p* < 0.05.

**Figure 3:**
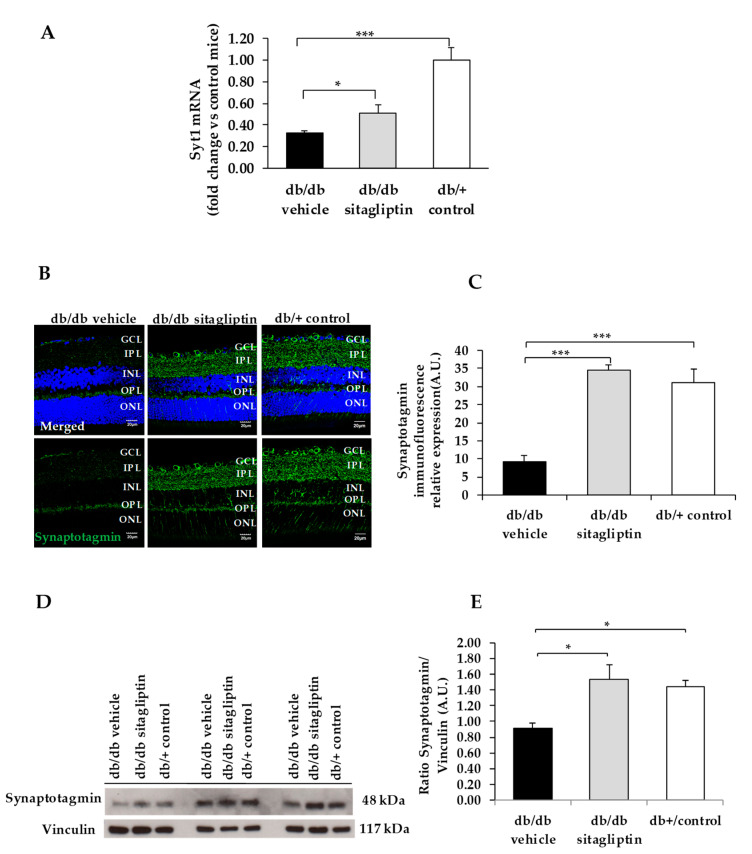
(**A**) Real-time quantitative RT-PCR analysis of synaptotagmin gene *(Syt1)* in db/db mice treated with vehicle (black bars), sitagliptin eye drops (grey bars), and in non-diabetic mice (white bars). (**B**) Immunofluorescence of synaptotagmin (green) among representative samples of diabetic retinas of experimental groups. Hoechst staining (blue) was used for nuclei labeling. ONL: outer nuclear layer; OPL: outer plexifom layer; INL: inner nuclear layer; IPL: inner plexiform layer; GCL: ganglion cell layer. Scale bars, 20 µm. (**C**) Quantification of synaptotagmin immunofluoresence in diabetic mice treated with vehicle eye drops (black bars), sitagliptin eye drops (grey bars), and in non-diabetic retinas (white bars). (**D**) Western blot bands of synaptotagmin, and (**E**) Densitometric analysis in db/db mice treated with vehicle eye drops (black bars), sitagliptin eye drops (grey bars), and in non-diabetic mice retinas (white bars). Protein levels were normalized with vinculin. * *p* < 0.05, *** *p* < 0.001.

**Figure 4:**
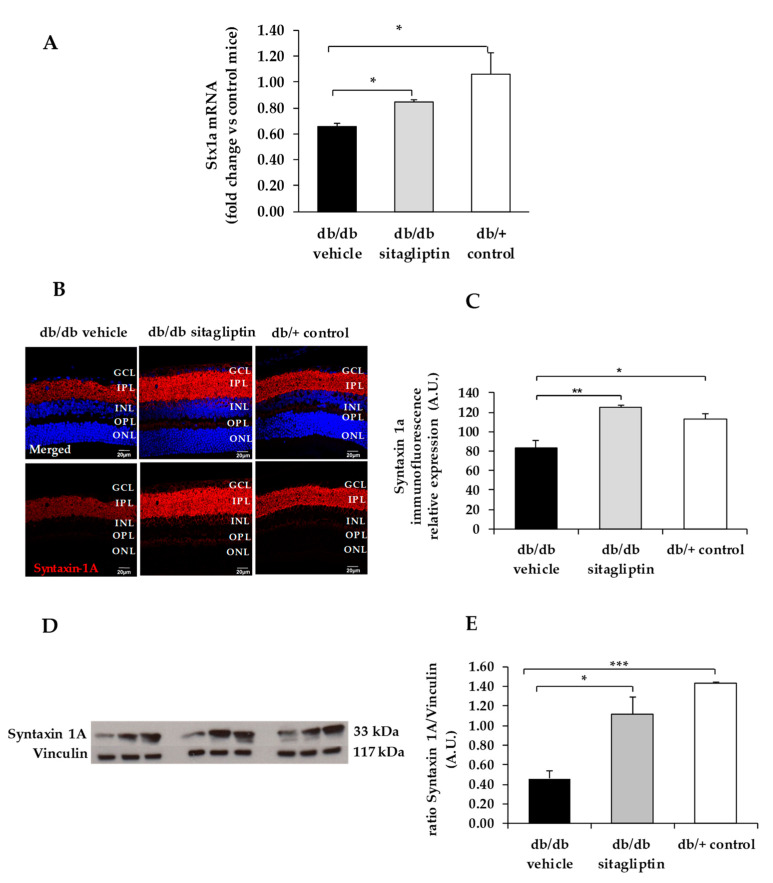
(**A**) Real-time quantitative RT-PCR analysis of syntaxin 1A gene *(Stx1a)* in db/db mice treated with vehicle (black bars), sitagliptin eye drops (grey bars), and in non-diabetic mice (white bars). (**B**) Immunofluorescence of syntaxin 1A (red) among representative samples of diabetic retinas of experimental groups. Hoechst staining (blue) was used for nuclei labeling. ONL: outer nuclear layer; OPL: outer plexifom layer; INL: inner nuclear layer; IPL: inner plexiform layer; GCL: ganglion cell layer. Scale bars, 20 µm. (**C**) Quantification of syntaxin 1A mmunofluorescence in diabetic mice treated with vehicle eye drops (black bars), sitagliptin eye drops (grey bars), and in non-diabetic retinas (white bars). (**D**) Western blot bands of syntaxin 1A and (**E**) Densitometric analysis in db/db mice treated with vehicle eye drops (black bars), sitagliptin eye drops (grey bars), and in non-diabetic mice retinas (white bars). Protein levels were normalized with vinculin. * *p* < 0.05, ** *p* < 0.01, *** *p* < 0.001.

**Figure 5:**
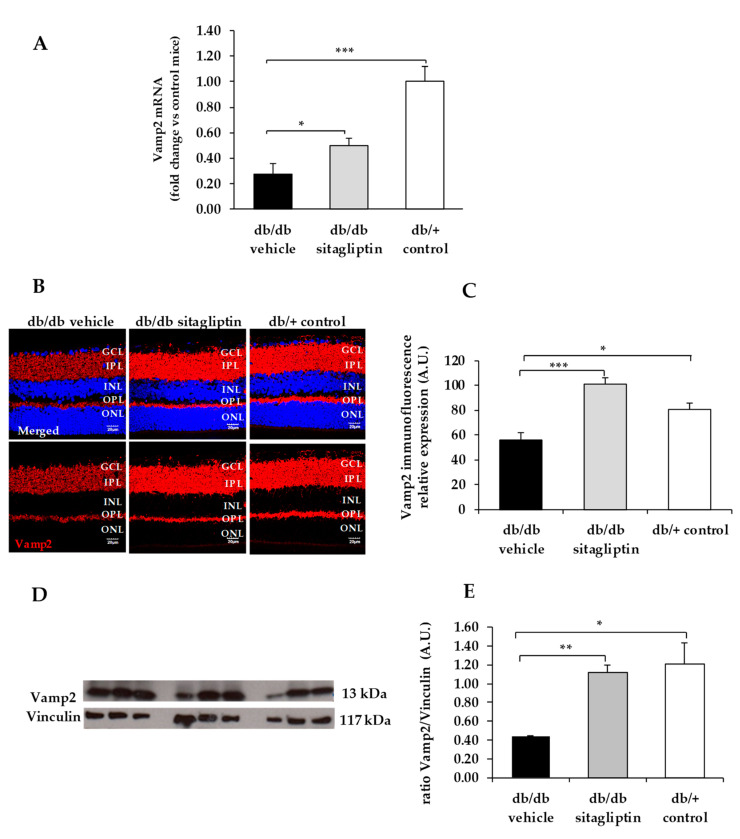
(**A**) Real-time quantitative RT-PCR analysis of VAMP2 gene *(Vamp2)* in db/db mice treated with vehicle (black bars), sitagliptin eye drops (grey bars), and in non-diabetic mice (white bars). (**B**) Immunofluorescence of VAMP2 (red) among representative samples of diabetic retinas of experimental groups. Hoechst staining (blue) was used for nuclei labeling. ONL: outer nuclear layer; OPL: outer plexifom layer; INL: inner nuclear layer; IPL: inner plexiform layer; GCL: ganglion cell layer. Scale bars, 20 µm. (**C**) Quantification of VAMP2 immunofluoresence in diabetic mice treated with vehicle eye drops (black bars), sitagliptin eye drops (grey bars), and in non-diabetic retinas (white bars). (**D**) Western blot bands of VAMP2 and (**E**) Densitometric analysis in db/db mice treated with vehicle eye drops (black bars), sitagliptin eye drops (grey bars), and in non-diabetic mice retinas (white bars). Protein levels were normalized with vinculin. * *p* < 0.05, ** *p* < 0.01, *** *p* < 0.001.

**Figure 6:**
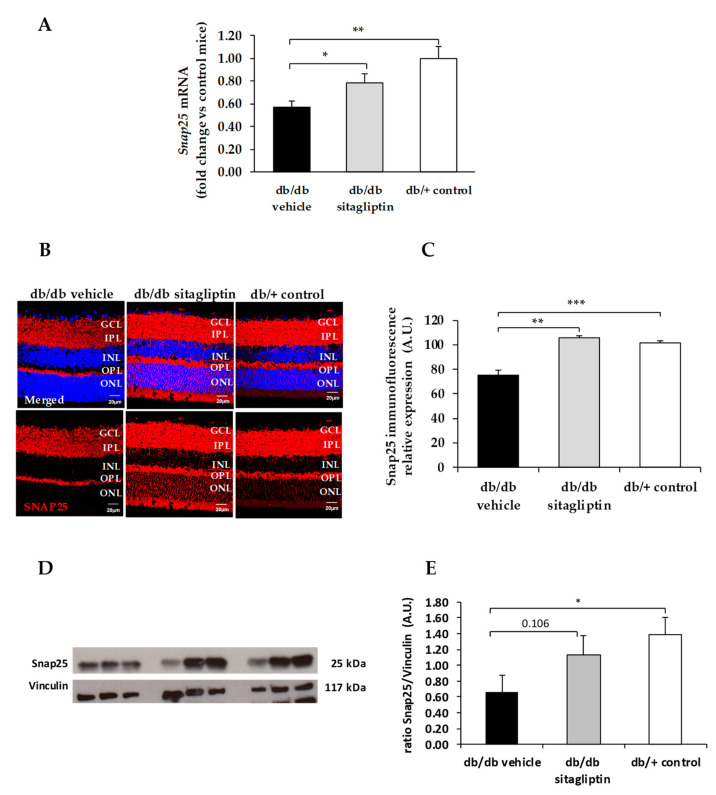
(**A**) Real-time quantitative RT-PCR analysis of SNAP25 gene *(Snap25)* in db/db mice treated with vehicle (black bars), sitagliptin eye drops (grey bars), and in non-diabetic mice (white bars). (**B**) Immunofluorescence of SNAP25 (red) among representative samples of diabetic retinas of experimental groups. Hoechst staining (blue) was used for nuclei labeling. ONL: outer nuclear layer; OPL: outer plexifom layer; INL: inner nuclear layer; IPL: inner plexiform layer; GCL: ganglion cell layer. Scale bars, 20 µm. (**C**) Quantification of SNAP25 immunofluoresence in diabetic mice treated with vehicle eye drops (black bars), sitagliptin eye drops (grey bars), and in non-diabetic retinas (white bars). (**D**) Western blot bands of SNAP25 and (**E**) Densitometric analysis in db/db mice treated with vehicle eye drops (black bars), sitagliptin eye drops (grey bars), and in non-diabetic mice retinas (white bars). Protein levels were normalized with vinculin. * *p* < 0.05, ** *p* < 0.01, *** *p* < 0.001.

We found a downregulation (protein and mRNA levels) of several presynaptic proteins such as synapsin I (Figure 1), synaptophysin (Figure 2), synaptotagmin (Figure 3), syntaxin 1A (Figure 4), vesicle-associated membrane protein 2 (VAMP2) (Figure 5), and synaptosomal-associated protein of 25 kDa (SNAP25) (Figure 6) in db/db mice treated with vehicle in comparison with non-diabetic mice. All these presynaptic proteins are involved in vesicle biogenesis, mobilization and docking, membrane fusion and recycling, and synaptic neurotransmission [15].

**Figure 7:**
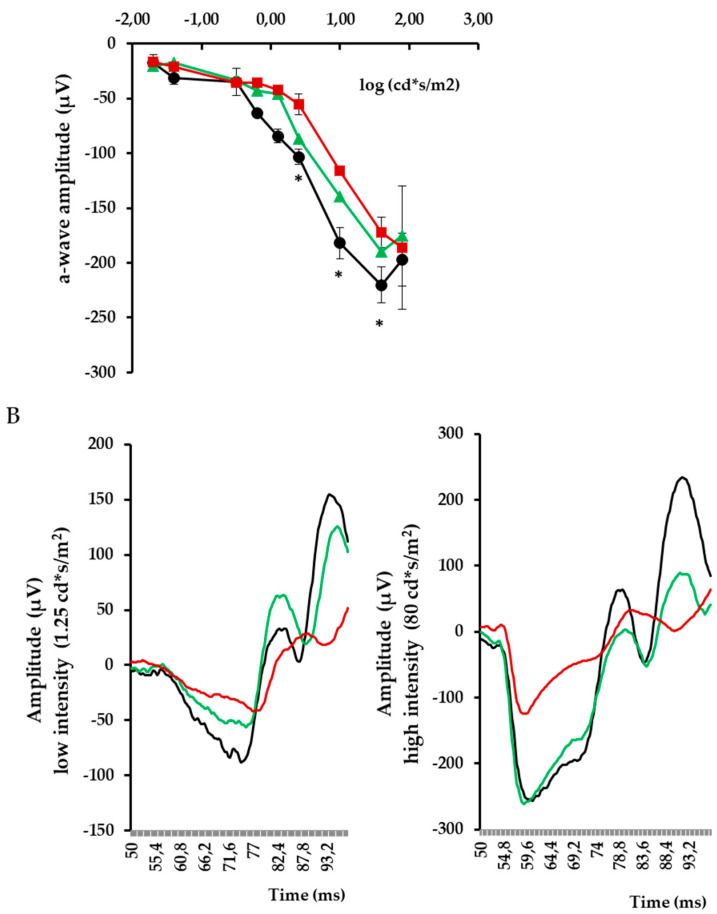
(**A**) Quantitative analyses of a wave amplitude in db/db mice treated with vehicle (red), db/db mice treated with sitagliptin (green), and non-diabetic mice (black). * *p* < 0.05 vs. db/db mice treated with vehicle. (**B**) Electroretinogram traces in response to low and high stimulus intensities in a representative non-diabetic mouse (black), a db/db mouse treated with vehicle (red), and a db/db mouse treated with sitagliptin (green).

In addition, we found that topical administration of sitagliptin was able to prevent the downregulation of all these synaptic proteins (Figure 1, Figure 2, Figure 3, Figure 4, Figure 5 and Figure 6) in diabetic mice. These effects were associated with an improvement of functional abnormalities induced by diabetes assessed by full-field ERG (Figure 7). The amplitude of a wave (predominantly produced by photoreceptors cells) was significantly lower in diabetic mice treated with the vehicle than in non-diabetic mice (Figure 7A). An example of an electroretinogram in response to low and high stimulus intensities in a representative non-diabetic mouse, a db/db mouse treated with vehicle, and a db/db mouse treated with saxagliptin or sitagliptin is shown in Figure 7B.

**Figure 8:**
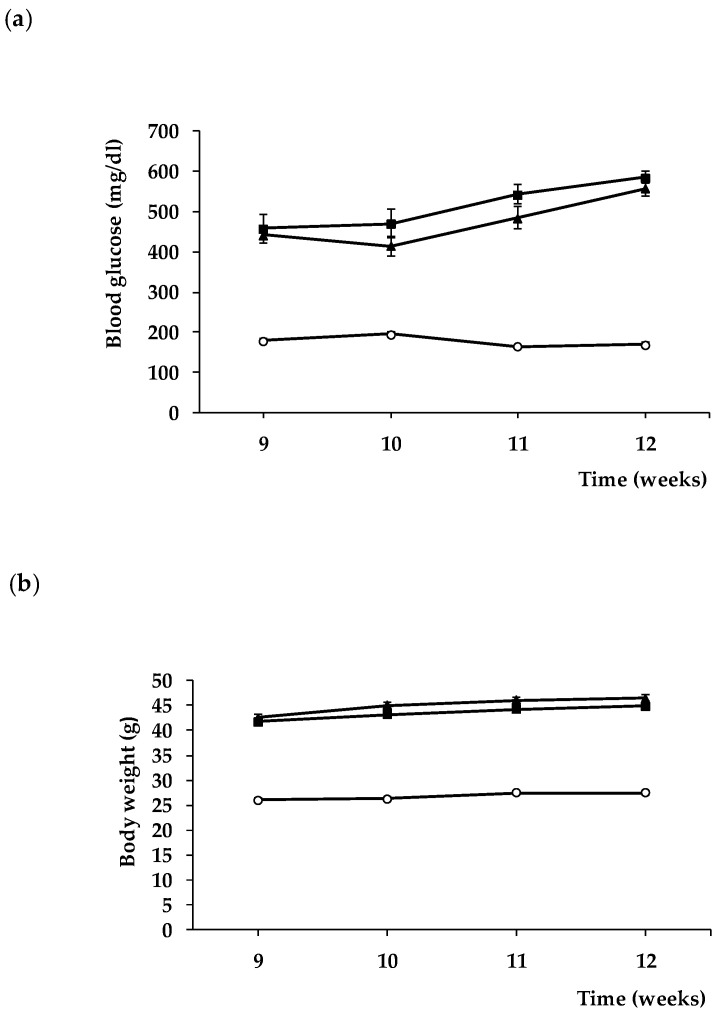
Evolution of blood glucose (**a**) and body weight (**b**) in db/+ mice (white circles), db/db mice treated with vehicle (black squares), and db/db mice treated with sitagliptin eye drops (black triangles).

It is worth mentioning that blood glucose concentrations and body weight during treatment were similar in db/db mice treated with sitagliptin and in db/db mice treated with vehicle (Figure 8). These findings indicate that the observed effects of sitagliptine are due to direct effect on the retina rather than a result of an improvement of systemic metabolic control.

## 4. Discussion

In the present study we provide evidence that topical ocular administration of sitagliptin (eye drops) was able to prevent the downregulation of presynaptic proteins in retinal neurons in db/db mice. This neuroprotective effect was associated with the improvement of retinal function assessed by ERG.

Synapsin and synaptophysin are the most abundant synaptic vesicle proteins and they are the most commonly used markers for presynaptic terminals [16]. Synapsin is the major peripheral membrane protein accounting for 6% of the total synaptic vesicle protein [17]; it regulates the reserve pool of synaptic vesicles available for exocytosis [18] and maintains the organization and abundance of vesicles at presynaptic terminals [19]. Synaptophysin is the major integral membrane protein accounting for 6–10% of total synaptic vesicle protein [20,21,22], which regulates the kinetics of synaptic vesicle endocytosis [23] as well as the synaptic vesicle retrieval through its interaction with VAMP2 [24]. SNAP25, VAMP2, syntaxin, and synaptotagmin are SNARE proteins (soluble N-ethylmaleimide-sensitive fusion protein attachment protein receptors) which regulate the synaptic vesicles exocytosis and neurotransmitter release [25].

Synaptophysin was the first synaptic vesicle protein to be cloned and since its discovery in 1985 [20,21] has been the most used marker to study the distribution of synapses. Masser et al. reported that diabetes causes a reduction in the retinal content of synaptophysin in STZ-induced diabetic rats, and this abnormality was corrected by insulin treatment when it was initiated soon after the onset of diabetes [26]. By contrast, when insulin was given later (i.e., after 1.5 months of uncontrolled diabetes), the synaptic protein content was not returned to control levels [26]. The authors concluded that retinal synapses were lost within 1 month of uncontrolled diabetes and suggest that synapses are not regained with glycemic control and restoration of insulin signaling. We found that the reduction of synaptophysin, as well as other relevant synaptic proteins also occurs in db/db mouse, a type 2 diabetic model with hyperinsulinemia and insulin resistance. In addition, and most importantly, we provide evidence that sitagliptin was able to restore synaptophysin and the other synaptic proteins after several weeks of significant hyperglycemia without any change in systemic blood glucose levels. Therefore, the effects of sitagliptin were due to its direct effects in the retina and unrelated to the improvement of the systemic diabetic milieu.

Regarding the mechanisms involved in the neuroprotective action of sitagliptin, we previously demonstrated that sitagliptin increases retinal content of GLP-1, a neuroprotective peptide for the retina [27], by inhibiting its degradation [12]. In this regard, it has been reported that sitagliptin regulates GABAergic transmission via the GLP-1/GLP-1R pathway [28]. Moreover, we found that sitagliptin prevents the increase of glutamate induced by diabetes by inhibiting GLAST downregulation and had also a strong anti-inflammatory action [12]. Although the main actions of sitagliptin seem mediated by an increase of retinal GLP-1 levels, the simultaneous activation of other mechanisms unrelated to GLP-1/GLP-1R cannot be ruled out [29].

It should be noted that DPP4 has been found embedded in synaptophysin stained elements, thus suggesting a close relationship between synaptic and DPP4 activity in the brain [30]. In addition, inflammation causes a five-fold increase in DPP4 protein level, suggesting a very potent posttranscriptional control of DPP4 expression during developing inflammation [30]. Given that the retina is ontogenically a brain-derived tissue, it could be expected that similar findings will be expected in retinas. Our data support this hypothesis given that we found that the inhibition of DPP4 by sitagliptin has a particular role in the presynaptic protein expression.

The global prevalence of DR in the population with diabetes is around one-third and approximately one-tenth of these patients have the vision-threatening states [31]. In addition, the number of people with visual impairment owing to DR worldwide is rising and this represents an increasing proportion of all causes of blindness and moderate or severe vision impairment [32]. In early stages of DR, aggressive treatments such as laser photocoagulation or intravitreal injections of corticosteroids or anti-VEGF agents are inconceivable. For this purpose, it is desirable to achieve a topical administration (i.e., eye drops) of therapeutic agents, such as sitagliptin. Apart from allowing self-administration, the topical administration of sitagliptin can also limit its action to the eye and minimize the associated systemic effects. Furthermore, it should be noted that is not proved that sitagliptin systemically administered can cross the blood–retinal barrier. Therefore, for achieving the direct beneficial effects of sitagliptin, the ocular topical route seems mandatory.

In summary, we conclude that sitagliptin exerts beneficial effects in the retinas of diabetic mice by preventing the downregulation of crucial presynaptic proteins. This effect can be added to the mechanisms involved in neuroprotective action of sitagliptin, an open and new avenue for treating DR as well as other retinal diseases in which neurodegeneration plays a relevant role.

## Figures and Tables

**Table 1:** Primers used for RT-PCR.

Primers		Nucleotide Sequence
*B2m*	Forward (5′-3′)	5′-GTATGCTATCCAGAAAACCC-3′
	Reverse (5′-3′)	5′-CTGAAGGACATATCTGACATC-3′
*Actb*	Forward (5′-3′)	5′-CTAAGGCCAACCGTGAAAG -3′
	Reverse (5′-3′)	5′-CAGTATGTTCGGCTTCCCATTC-3′
*Syn1*	Forward (5′-3′)	5′-AATCACAAAGAGATGCTCAG-3′
	Reverse (5′-3′)	5′-GGACACGCACATCATATTTAG-3′
*Syp*	Forward (5′-3′)	5′-TGCCAACAAGACGGAGAGTG-3′
	Reverse (5′-3′)	5′-TAGTGCCCCCTTTAACGCAG-3′
*Syt1*	Forward (5′-3′)	5′-ACCCTGGGCTCTGTATCCC-3′
	Reverse (5′-3′)	5′-CCCTGACCACTGAGTGCAAA-3′
*Stx1a*	Forward (5′-3′)	5′-CGCTGTCCCGAAAGTTTGTG-3′
	Reverse (5′-3′)	5′-GTGTCTGGTCTCGATCTCACT-3′
*Vamp2*	Forward (5′-3′)	5′-ATCATCGTTTACTTCAGCAC-3′
	Reverse (5′-3′)	5′-TGAAAGATATGGCTGAGAGG-3′
*Snap25*	Forward (5′-3′)	5′-CAACTGGAACGCATTGAGGAA-3′
	Reverse (5′-3′)	5′-GGCCACTACTCCATCCTGATTAT-3′

**Table 2:** Primary antibodies used for Western blotting.

Primary Antibodies	Description
Synapsin I	Rabbit polyclonal; 1:2000; ab64581; Abcam, Cambridge, UK
Synaptophysin	Rabbit monoclonal; 1:200,000; ab32127; Abcam, Cambridge, UK
Synaptotagmin	Mouse monoclonal; 1:1,000; ab13259; Abcam, Cambridge, UK
Syntaxin 1A	Rabbit polyclonal; 1:100,000; ab41453; Abcam, Cambridge, UK
SNAP-25	Rabbit polyclonal; 1:1000; 14903-1-AP; Proteintech, Rosemont, IL, USA
Vamp2	Rabbit polyclonal; 1:1000; 10135-1-AP; Proteintech, Rosemont, IL, USA
Cyclophilin A	1:10,000; BML-SA296; Enzo, NY, USA
Vinculin	Mouse monoclonal; 1:7000; sc-73614; Santa Cruz, Dallas, TX, USA

**Table 3:** Primary and secondary antibodies used for immunofluorescence experiments.

**Primary Antibodies**	**Description**
Synapsin I	Rabbit polyclonal; 1:100; ab64581; Abcam, Cambridge, UK
Synaptophysin	Rabbit monoclonal; 1:100; ab32127; Abcam, Cambridge, UK
Synaptotagmin	Mouse monoclonal; 1:200; ab13259; Abcam, Cambridge, UK
Syntaxin 1A	Rabbit polyclonal; 1:200; ab41453; Abcam, Cambridge, UK
Vamp2	Rabbit polyclonal; 1:100; 10135-1-AP; Proteintech, Rosemont, IL, USA
SNAP-25	Rabbit polyclonal; 1:100; 14903-1-AP; Proteintech, Rosemont, IL, USA
**Secondary Antibodies**	**Description**
Alexa Fluor 488 Goat anti-mouse	Goat polyclonal; 1:600; ab150113; Abcam, Cambridge, UK
Alexa Fluor 488 Goat anti-rabbit	Goat polyclonal; 1:600; ab150081; Abcam, Cambridge, UK
Alexa Fluor 594 Goat anti-mouse	Goat polyclonal; 1:600; A-11032; ThermoFisher Scientific, Waltham, MA, USA
Alexa Fluor 594 Goat anti-rabbit	Goat polyclonal; 1:600; A-11012; ThermoFisher Scientific, Waltham, MA, USA

## Data Availability

The data presented in this study are available on request from the corresponding author.
